# Fabrication of a lead-free ternary ceramic system for high energy storage applications in dielectric capacitors

**DOI:** 10.3389/fchem.2022.1025030

**Published:** 2022-10-19

**Authors:** Azam Khan, Noor Shad Gul, Mao Luo, Jianbo Wu, Shahan Zeb Khan, Abdul Manan, Xiu-Jian Wang, Taj Malook Khan

**Affiliations:** ^1^ School of Chemistry and Pharmacy, Guangxi Normal University, Guilin, China; ^2^ Drug Discovery Research Center, Southwest Medical University, Luzhou, China; ^3^ Department of Pharmacology, Laboratory for Cardiovascular Pharmacology, School of Pharmacy, Southwest Medical University, Luzhou, China; ^4^ Department of Chemistry, University of Science and Technology, Bannu, Pakistan; ^5^ Advanced Materials Research Laboratory, Department of Physics, University of Science and Technology Bannu, Bannu, Pakistan

**Keywords:** perovskite, recoverable energy, pulsed power, dielectric capacitors, temperature

## Abstract

The importance of electroceramics is well-recognized in applications of high energy storage density of dielectric ceramic capacitors. Despite the excellent properties, lead-free alternatives are highly desirous owing to their environmental friendliness for energy storage applications. Herein, we provide a facile synthesis of lead-free ferroelectric ceramic perovskite material demonstrating enhanced energy storage density. The ceramic material with a series of composition (1-z) (0.94Na_0.5_Bi_0.5_TiO_3_-0.06BaTiO_3_)-zNd_0.33_NbO_3,_ denoted as NBT-BT-zNN, where, z = 0.00, 0.02, 0.04, 0.06, and 0.08 are synthesized by the conventional solid-state mix oxide route. Microphases, microstructures, and energy storage characteristics of the as-synthesized ceramic compositions were determined by advanced ceramic techniques. Powder X-ray diffraction analysis reveals pure single perovskite phases for z = 0 and 0.02, and secondary phases of Bi_2_Ti_2_O_7_ appeared for z = 0.04 and 0.08. Furthermore, scanning electron microscopy analysis demonstrates packed-shaped microstructures with a reduced grain size for these ceramic compositions. The coercive field (E_c_) and remnant polarization (P_r)_ deduced from polarization vs. electric field hysteresis loops determined using an LCR meter demonstrate decreasing trends with the increasing z content for each composition. Consequently, the maximum energy storage density of 3.2 J/cm^3^, the recoverable stored energy of 2.01 J/cm^3^, and the efficiency of 62.5% were obtained for the z content of 2 mol% at an applied electric field of 250 kV/cm. This work demonstrates important development in ceramic perovskite for high power energy storage density and efficiency in dielectric capacitors in high-temperature environments. The aforementioned method makes it feasible to modify a binary ceramic composition into a ternary system with highly enhanced energy storage characteristics by incorporating rare earth metals with transition metal oxides in appropriate proportions.

## Introduction

The world leaders have signed the Paris Agreement on climate change to fix global warming <1.5°C by reducing the emission of greenhouse gases by the end of 2030 and reaching zero levels by 2050 (Vicedo-Cabrera et al., 2018). In recent years, the emission of CO_2_ has increased immensely in the upper atmosphere due to the burning of fossil fuels for transportation, domestic uses, and industrial developments. Hence, this tremendous amount of CO_2_ has seriously impaired the Earth’s environment due to the greenhouse effect that caused global warming, climate change, and acidification of oceanic water (Letcher, 2020; Chen et al., 2022). The feasible solution for both these issues of global warming and climate change is to substitute fossil fuel energy sources with renewable energy sources such as sunlight, tides, waves, and wind power as clean and free of greenhouse gases (Panwar et al., 2011; Lima et al., 2020). The energy produced from these inexhaustible energy resources needs energy storage materials and devices to store it for future use without disturbing the Earth’s environment (Timmons et al., 2014; Krishan & Suhag, 2019). In this regard, dielectric materials with high energy storage density can be used to miniaturize the component employed in most electronic devices (Kyeremateng et al., 2017; Liu et al., 2019). Currently, most energy storage technologies are based on batteries, fuel cells, supercapacitors, and dielectric capacitors (Winter & Brodd, 2004; Kim et al., 2015; Wu et al., 2019). Compared to batteries and fuel cells, capacitors are highly cost-effective, stable thermally, and robust, working with high power density. Numerous dielectric capacitors are based on polymers and ceramics with high output power, fast charge–discharge rate, and long working life in high-temperature environments (Fan et al., 2018; Palneedi et al., 2018; Zhou et al., 2018). Dielectric ceramic-based capacitors are superior to polymer-based dielectrics owing to their stable energy potential and working at a broader temperature range. However, dielectric ceramics have a drawback of low energy storing density compared to dielectric polymers because of their low dielectric breakdown strength (DBS), which reduces their range of applications (Zeb & Milne, 2015; Li Q et al., 2018; Zaman, et al., 2021a). Thus, developing new dielectric ceramic systems with high energy storage density is more valuable for miniaturization, integration, and lightweight energy storage devices under high-temperature conditions (Jiang et al., 2021; Yang et al., 2019; Zaman, et al., 2022a).

Typically, ceramic materials applied for dielectric capacitors have linear dielectric, ferroelectric, anti-ferroelectric, and relaxor ferroelectric characteristics (Zheng et al., 2021; Zhou et al., 2020). The recoverable energy density (W_rec_) is deduced from the energy storage ability of dielectric substances. The recoverable and storage energy densities are evaluated mathematically in Eqs 1,2 (Wang et al., 2019).

$$W_{s}=\int_{0}^{P_{\max}}EdP$$ (1)

$$W_{rec}=\int_{P_{r}}^{P_{\max}}EdP$$ (2)

The efficiency can be determined from Eq. 3 as follows:

$$\eta =W_{res}/W_{s}\times 100\;\%$$ (3)

The aforementioned equations show that maximum polarization (P_max_), high permittivity, large DBS, and low remnant polarization (P_r_) are crucial for dielectric ceramics to achieve maximum storage power density and high efficiency. In this regard, an enormous amount of research has been carried out for lead-containing dielectric ceramics owing to their high energy storage characteristics such as (Pb, La)ZrO_3_ (PLZ), Pb(Zr, Sn, Ti)O_3_ (PZST), and (Pb, La) (Zr, Ti)O_3_ (PLZT) (Parui & Krupanidhi, 2008; Jia et al., 2018). However, lead-consistent ceramics are toxic and have an environmental impact; therefore, lead-free ceramic compositions with high energy storage characteristics must be fabricated. In this regard, a lead-free ceramic composition Na_0.5_ Bi_0.5_TiO_3_ (NBT) has received greater attention since it has a large maximum polarization (P_max_∼40 μC/cm^2^) and high Curie temperature (T_c_∼320°C); however, it has large remnant polarization (P_r_) and high coercive field (E_c_) as well which make it unsuitable for energy storage applications (Li K et al., 2018; Ranjan, 2020). Therefore, massive research has been carried out in fabricating binary or ternary solid solutions of NBT by modifying it with Bi_0.5_ K_0.5_TiO_3_ (KBT), K_0.5_Na_0.5_NbO_3_ (KNN), and BaTiO3 (BT) for enhancing its energy storage competencies (Quan et al., 2014; Wong et al., 2015; Zhao et al., 2016; Zaman, et al., 2022b). Hence, Takenaka et al. (1991) prepared NBT_1-x_BT (BNBT) by adding BaTiO_3_ and studied its morphotropic phase boundary (MPB), developed for x ∼ 6–7 mol% for the assurance of large ferroelectric and piezoelectric characteristics in comparison with lead-consistent ceramics. In the same way, Li et al. (2016) fabricated a ceramic composition of 0.94Bi0.5Na0.5TiO3-0.06BaTiO3-0.03CaZrO_3_ *via* adding CaZrO_3_ has displayed an energy storage density of W_s_ ∼ 0.70 J/cm^3^. Likewise, J. Wang and Chen, (2014) prepared a ceramic composition of 0.92Na_0.47_Bi_0.47_Ba_0.06_TiO_3_-0.08KNbO_3_ by adding KNbO_3_ which resulted in W_s_ ∼ 0.89 J/cm^3^. In the same way, Li et al. (2019) fabricated a modified ceramic composition of 0.85 [(Na_0.5_Bi_0.5_)_0.94_Ba_0.06_]TiO_3_-0.15BaSnO_3_ by adding BaSnO_3_, which demonstrated an enhanced W_s_ ∼ 1.20 J/cm^3^ and efficiency of 86.7%. In this connection, J. Ding et al. (2014) also achieved W_s_ ∼ 0.90 J/cm^3^ for 0.89Bi0.5Na0.5TiO3-0.06BaTiO3-0.05K0.5Na0.5NbO3. These results demonstrate that the appropriate cation addition of alkali, alkaline earth, and rare earth metals at either A or B sites of ABO_3_ structure of (Na_0.5_Bi_0.5_)_0.94_Ba_0.06_TiO_3_) has reduced its E_c,_ which resulted in enhanced energy storage characteristics of this composition. The addition of Nd_0.33_ NbO_3_ into the (Na_0.5_Bi_0.5_)_0.94_Ba_0.06_TiO_3_) system suppressed its ferroelectric loops with the decreasing remnant polarization, which resulted in increased energy storage competencies in this ceramic composition (Jain et al., 2021; Huang et al., 2022).

The present work focuses on modifying the ceramic composition of 0.94(Na_0.5_Bi_0.5_)TiO_3_-0.06BaTiO_3_ by introducing Nd_0.33_ NbO_3_ to investigate its effects on the coercive field and remnant polarization. The influences of Nd_0.33_ NbO_3_ addition on the aforementioned composition have been studied in energy storage density, recoverable energy, and efficiency as a function of z content under high-temperature conditions.

## Experimental protocols

Several methods are used for the fabrication of lead-free electroceramic compositions, such as mechanochemical synthesis, sol–gel method, and current-assisted sintering method; however, in the bulk of electroceramic fabrication, the most common preparation method is solid-state mix oxide synthesis, typically from metal oxide–carbonate mixtures, and sintering. We employed a conventional solid-state mix oxide method to synthesize (NBT-BT-zNN), (z = 0.00, 0.02, 0.04, 0.06, 0.08) ceramic compositions, using the reagents: BaCO_3_ (99.9%), Bi_2_O_3_ (99%), TiO_2_ (99.8%), Na_2_CO_3_ (99.8%), Nb_2_O_5_ (99%), and Nd_2_O_3_ (99.9%). These starting raw materials in powder forms were heated at 100°C for two days before weighing their stoichiometric amounts to remove the trapped water moisture from bodies. The reagents were weighed stoichiometrically and then ball-milled with ethanol in plastic containers using ZrO_2_ grinding balls for 24 h, resulting in the formation of slurries, which were then dried at 95°C temperature. The milled powder of each composition was heated for 2 h at 850°C and then re-ball-milled for 12 h and dried to get fine powders. A few drops of liquid polyvinyl alcohol (PVA) as a binder were mixed with the dried samples and ground for 10 min. After completing this process, each sample was pressed to make a pellet of 1 mm height at a force of 150 MPa in a steel die of 12 mm diameter. These pellets were heated for 2 h at 600°C to remove PVA content and finally sintered at 1,050°C, 1,150°C, and 1,175°C for 2 h. The green body pellets of each composition were sintered at different temperatures, such as 1,050, 1,150, and 1,175°C, for 2 h to get the optimum density of each composition. We measure the density of each composition at the aforementioned temperatures using the Archimedes principle. The results showed that the density was higher at 1,150°C for each ceramic composition, and each composition’s properties were determined for the optimally dense sintered ceramics at 1,150°C for 2 h.

At the maximum density, a pellet of each sample was crushed and converted to powders. The phase analysis was carried out for the sintered samples using a powder X-ray diffraction technique. Using an X-ray diffractometer, the data were recorded at a scan rate of 0.02°/min at 2θ from 10–80°. For the microstructural study, a maximally dense pellet of each sample was smoothly polished and then thermally etched, followed by a gold coating to avoid the charging effect during the electron beam interaction. The microstructure of each sample was checked using a scanning electron microscope.

Furthermore, both the surfaces of a thick pellet of 0.65 mm thickness of each ceramic composition sintered at 1,150°C were highly polished, followed by a silver coating, and heated at 800°C for 2 h. The dielectric constant and tangent loss in each sample on 400, 550, 800, and 1 MHz vs. temperature were noted with a scan rate of 3°C/min using the LCR meter (Agilent E4980 A). The thickness of maximum dense samples was minimized up to 0.15 µm, then coated with silver films, and heated at 800°C for 2 h. Polarization vs. electric field (P-E) loops at breakdown voltage were taken at 10 Hz using a ferroelectric test method.

## Results and discussion

### Phases and microstructures interpretation

**FIGURE 1:**
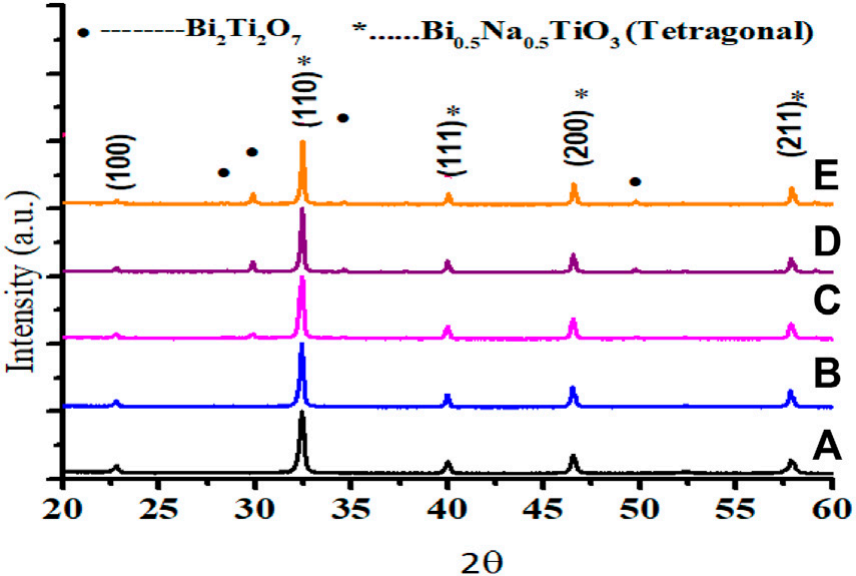
X-ray diffraction (XRD) patterns for (NBT-BT-zNN) **(A)** z = 0.00, **(B)** z = 0.02, **(C)** z = 0.04, **(D)** z = 0.06, and **(E)** z = 0.08.

Figure 1 shows typical PXRD patterns of ceramic compositions (1-z) (NBT-BT-zNN), (z = 0.00, 0.02, 0.04, 0.06, 0.08) recorded at room temperature with 2*θ* = 20–60°. The observed peaks labelled as “*” in XRD patterns were matched with PDF (070–4760), a single perovskite phase for z = 0.00 and z = 0.02, demonstrating complete diffusion of Nd_0.33_NbO_3_ into (NBT-BT) lattices and resulting in a slight expansion due to which (111) and (200) peaks have moved to a lower angle with the increasing content of Nd_0.33_NbO_3_. At the same time, a few low-intensity peaks labelled as “•” also appeared for z ≥ 0.04. These peaks matched with PDF (089–4732) and labelled as “•” exhibited the secondary phase (Bi_2_Ti_2_O_7_). The developments of the secondary phase indicate the solid solubility limit of Nd_0.33_NbO_3_ in the 0.94Bi_0.5_Na_0.5_TiO_3_-0.06BaTiO_3_ system of about 2 mol%. Previous studies also demonstrated the appearance of this Bi_2_Ti_2_O_7_ secondary phase in Bi_0.5_Na_0.5_TiO_3_-BaTiO_3_-based ceramics (Ding et al., 2022; Lu et al., 2011; Ni et al., 2012; Wang et al., 2022). This secondary phase Bi_2_Ti_2_O_7_ appeared due to the vaporization of Bi and Na at elevated temperatures (Xu et al., 2015). Furthermore, the value of Bi_2_Ti_2_O_7_ has increased with the increasing z content to 8 mol% in this current formulation.

**FIGURE 2:**
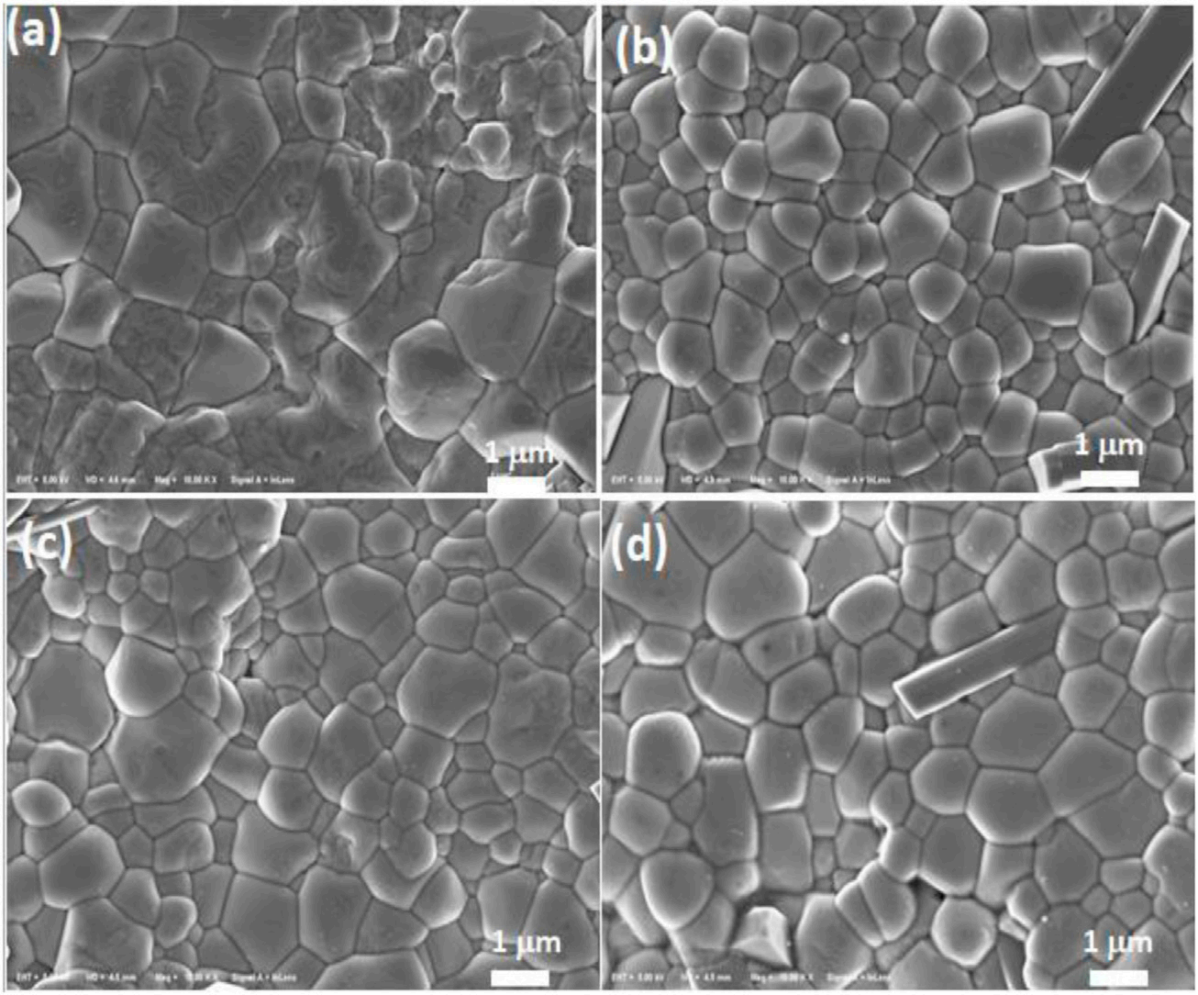
Scanning electron micrographs from thermally etched surfaces for (NBT-BT-zNN) **(A)** z = 0.00, **(B)** z = 0.02, **(C)** z = 0.04, and **(D)** z = 0.08.

Figure 2 displays the scanning electron micrographs of (NBT-BT-zNN), z = 0.00, 0.02, 0.04, and 0.08). There are clear grain boundaries observed for all the compositions with dense microstructures. Grain sizes are decreased with increasing z contents of z ≤ 0.06 and then slightly increased to the z content of 0.08. Several factors, including the liquid phase, dopants, and pore/voids, could affect the grain growth (Xu et al., 2017; H. Yang et al., 2017; Z. Yang et al., 2016). The increasing amount and ionic radii of Nb^5+^ (0.64 Å) against Ti^4+^ (0.60 Å) may also result in lower ionic mobility upon sintering and hence hinder the diffusion of ions that resulted in the slowdown of grain growth (Zaman et al., 2021). The regular distribution of grains is highly effective in increasing density and enhancing the DBS of dielectric ceramics (Begum et al., 2021; Fernandez-Benavides et al., 2018; Li et al., 2021). Some plates like elongated grains is observed in the microstructures reported in the previous literature (Wood et al., 1999).

### Dielectric characteristics

**FIGURE 3:**
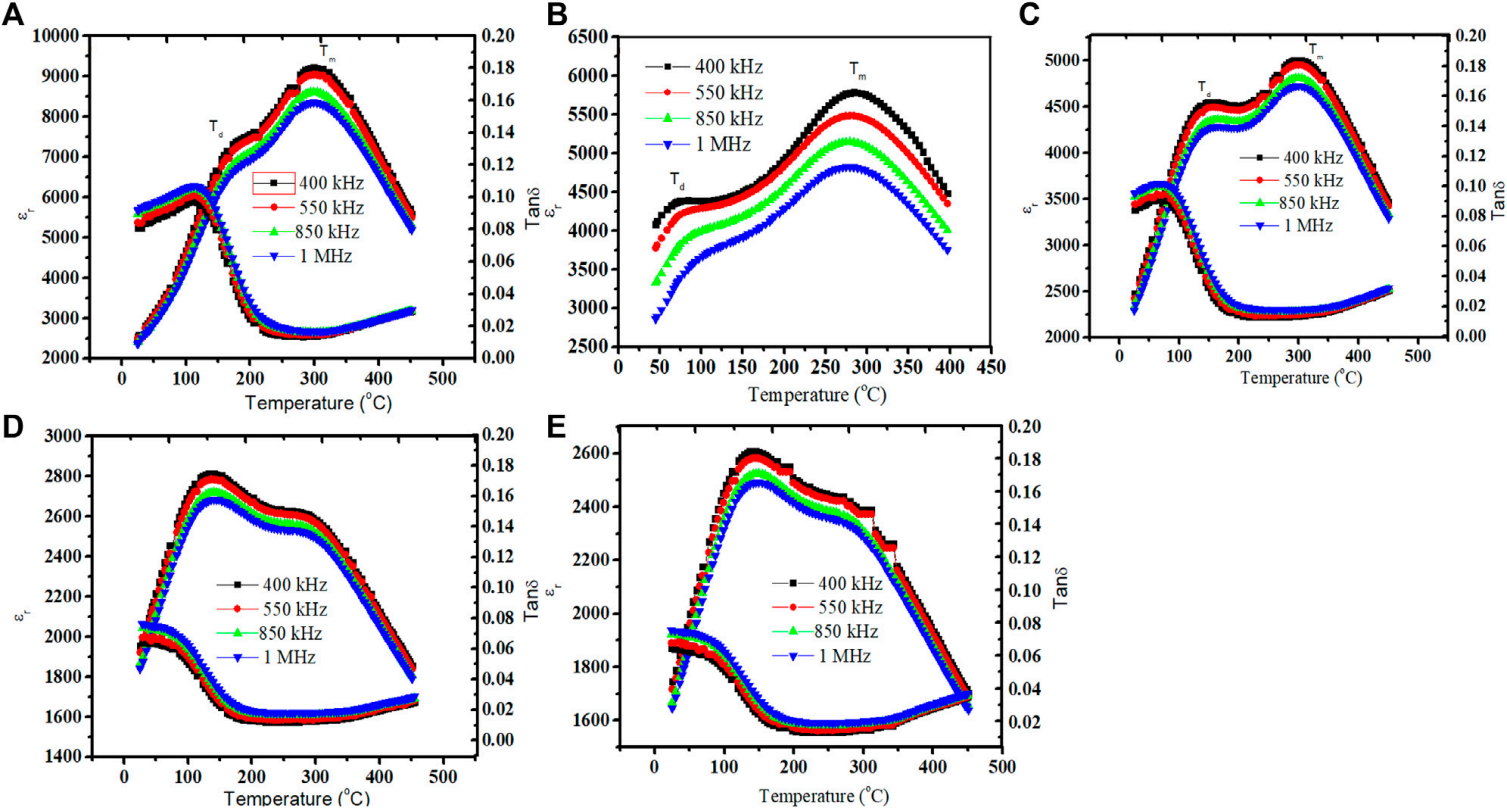
Changes in ε_r_ and tan δ measured at different frequencies for (NBT-BT-zNN) **(A)** z = 0.00, **(B)** z = 0.02, **(C)** z = 0.04, **(D)** z = 0.06, and **(E)** z = 0.08 with changes in temperature.

Figure 3 depicts the temperature-dependent variation of the relative permittivity (ε_r_) and tan *δ* (loss of the energy rate, also known as the dissipation factor) for (NBT-BT-zNN), (z = 0.00, 0.02, 0.04, and 0.08) ceramics measured at 400 kHz, 550 kHz, 850 kHz, and 1 MHz with a temperature of 25–500°C. Two peaks were observed in each ɛ_r_ curve with temperature variation. The peak at a lower temperature is called the depolarization temperature (T_d_), and the other peak (T_m_) shows the maxima of the dielectric constant at high temperature, called the Curie temperature. These two anomalies are also observed in BNT ceramics (Trolliard & Dorcet, 2008; Jo et al., 2011; Butnoi et al., 2018). The anti-ferroelectric-to-paraelectric phase transformation is observed across the Curie temperature (T_m_) (Pan et al., 2019). A frequency dispersion and phase transition peak of the ferroelectric-to-paraelectric phase of (NBT-BT-zNN) ceramics demonstrated distorted long-range ordering of the ferroelectric phase to develop relaxor nature. Likewise, T_d_ has shifted toward high temperature upon the variation of frequency from 450 kHz to 1 MHz, indicating an increase in the relaxor behavior. Furthermore, T_d_ observed >150°C at 1 kHz but gradually moved to a lower temperature (<75°C) upon increasing the z content. The broadening of the ε_r_ versus T curve between T_d_ and T_m_ shows an improvement in the stability of energy storage characteristics with temperature. Furthermore, adding the z content has decreased T_m_ and ε_m_ of these ceramics (Dittmer et al., 2012; Pradhan et al., 2018; Jin, Li, & Zhang, 2020).

**FIGURE 4:**
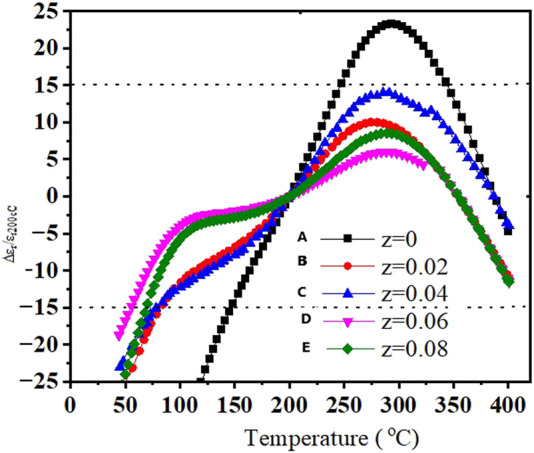
Changes in Δε_r_/ε_r200_°C for (NBT-BT-zNN) **(A)** z = 0.00, **(B)** z = 0.02, **(C)** z = 0.04, **(D)** z = 0.06, and **(E)** z = 0.08 ceramics with temperature at 1 MHz.

The stable nature of the ceramic’s dielectric constant with temperature is another important parameter. There are changes observed in permittivity (± 15%) in the temperature range of 44–400°C for (z = 0.00, 0.02, 0.04, and 0.08) ceramics, measured at 1 MHz using Eq. 4, as shown in Figure 4.

$$\Delta \varepsilon_{r}/\varepsilon_{r}200{}^{\circ}C,$$ (4)

where Δε_r_ = ε_r_T-ε_r_200°C. Δε_r_/ε_r200_°C for a sample of z = 0.02 displayed good thermal stability in the temperature range of 83–420°C. Similarly, z = 0.6 also displayed thermal stability in the temperature range of 55–430°C.

### Ferroelectricity and energy storage characteristics

**FIGURE 5:**
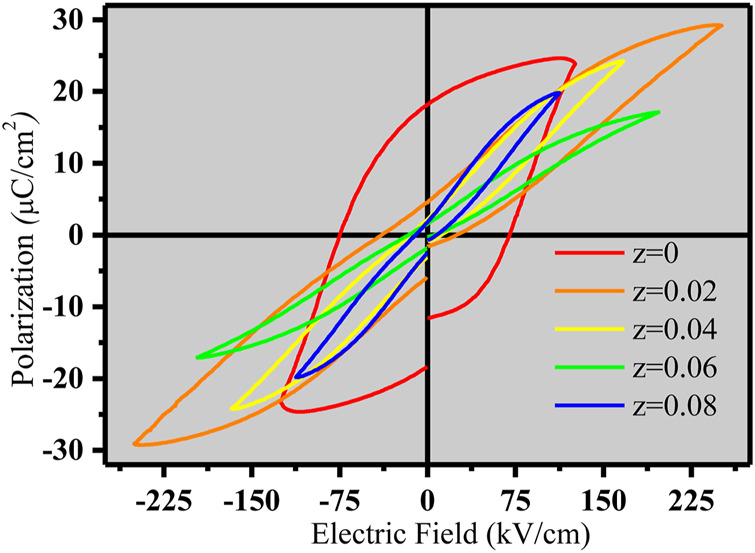
Polarization versus electric field (P-E) hysteresis loops of (1-z) (NBT-BT-zNN) ceramics.

The polarization vs. electric field (P–E) hysteresis loops with a frequency of 10 Hz were recorded at 25°C for (NBT-BT-zNN), (z = 0.00, 0.02, 0.04, and 0.08) ceramics, as shown in Figure 5. The observed P-E loop became slim with the increased z content, showing enhancement in the ferroelectric nature of the 0.94Na_0.5_Bi_0.5_TiO_3_-0.06BaTiO_3_ ceramic system. The increase in the z content has decreased the remnant polarization (P_r_), suggesting the Nb^5+^ substitution for Ti^4+^ at the B-site and Nd^3+^ for (Ba^2+^/Bi^3+^/Na^1+^) at the A-site disrupting the long-range ordering of domains and converting it to the polar nano-regions (PNRs) (Mayamae et al., 2016; Hu et al., 2018). The sizes of PNRs are small as compared to the ferroelectric micro-domain. The PNRs can easily be aligned back and forth by the applied electric field, which causes a decrease in the P_r_ value (Wen et al., 2018).

Figure 5 shows a double double-like P-E hysteresis loop for the (NBT-BT-zNN) ceramic system with a good anti-ferroelectric behavior for z = 0.02, which is very important for energy storage applications. This anti-ferroelectric behavior is also observed in the NBT-BT-La system (P. Fan et al., 2021; Liu et al., 2013). The doping of the z content decreased Pr, resulting in depolarization at room temperature. Keeping of the high-electric field has generated electric field-induced polarization that caused a decrease in P_r_ and an increase in the W_rec_ value. Thus, a decreased P_r_ value has increased the W_rec_ value for the (NBT-BT-zNN) ceramic system at 250 kV/cm for the z content of 2 mol%.

**FIGURE 6:**
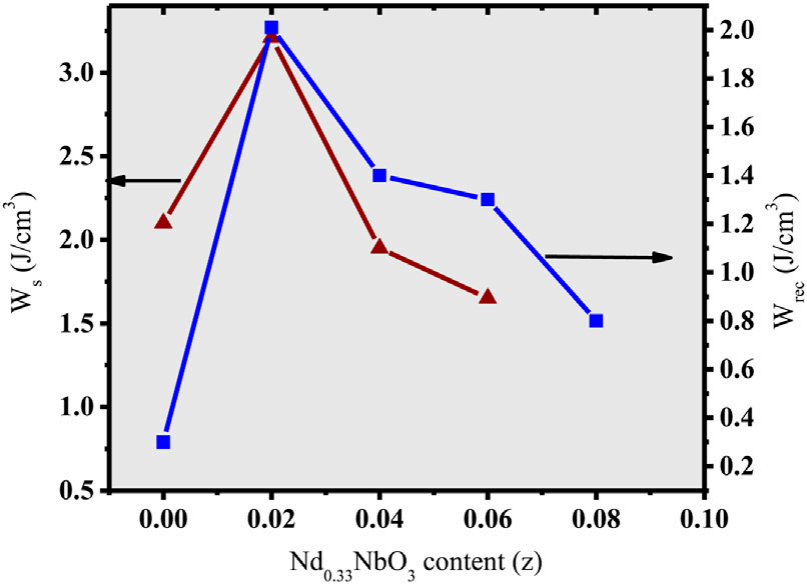
Changes in W_s_ and W_rec_ of (NBT-BT-zNN) ceramics with the z content.

**FIGURE 7:**
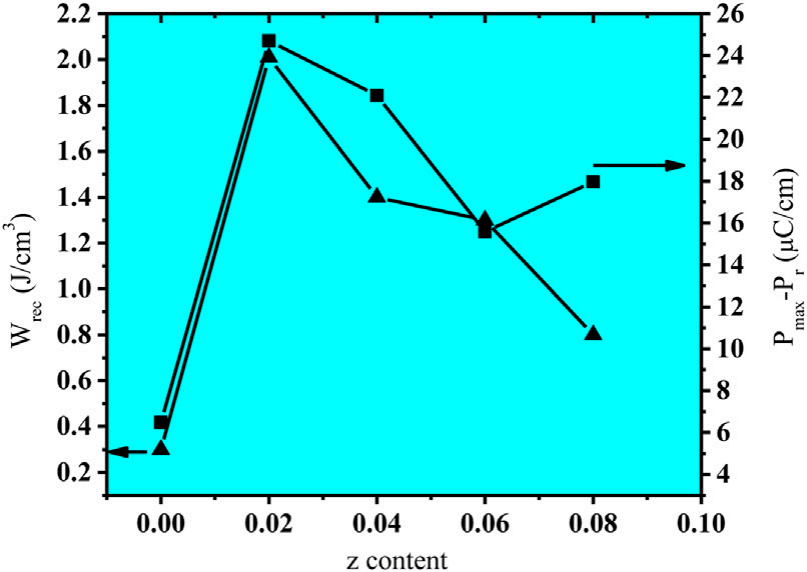
Changes in W_rec_ and P_max_-P_r_ for (NBT-BT-zNN) ceramics with the z content.

**FIGURE 8:**
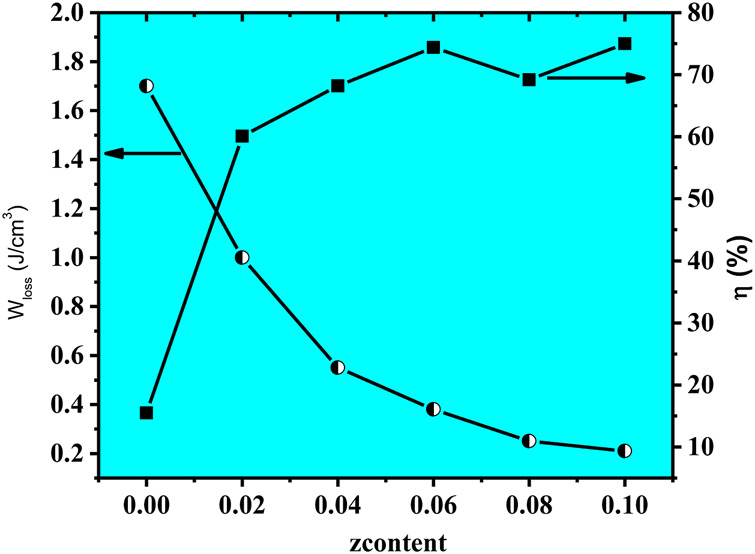
Changes in W_loss_ and η of (NBT-BT-zNN) ceramics with the z content.

The recoverable energy density (W_rec_) and energy storage density (W_s_) for the (NBT-BT-zNN) ceramic system is determined at the maximum applied electric field, as shown in Figure 6. The energy storage and recoverable energy density initially increased with the x content from 0 to 0.02 and then decreased with a further increase in the x content to 0.1. Both are related to the large and high breakdown strength. Furthermore, it is also reported that W_rec_ energy density strongly depends on P_max_-P_r_. The large value of P_mx_-P_r_ results into high W_rec_. Therefore, the high value of W_rec_ in the present study for x = 0.02 is also because of its large P_max_-P_r_ value compared to other x values, as shown in Figure 7. The large value of P_max_-P_r_ is related to its high breakdown strength and the lower remnant polarization. The increased breakdown electric field (E_b_) and large ∆P = P_max_-P_r_ value ensure a large value of W_rec_ for x = 0.02. Hence, W_s_∼3.2 J/cm^3^ along with W_rec_∼2.01 J/cm^3^ and efficiency of η∼62.5% were obtained for z = 0.02 at 250 kV/cm, as demonstrated in Figure 8. The large W_rec_ value is related to the anti-ferroelectric response induced in this parent formulation (0.94(Na_0.5_Bi_0.5_)TiO_3_-0.06BaTiO_3_) due to Nd_0.33_NbO_3_ doping up to 2 mol%.

## Conclusion

Capacitors based on relaxor dielectrics are promising candidates for pulsed power applications as they have high energy storage capabilities, high output power, fast charging–discharging rate, and good electric breakdown strength performances under high-temperature conditions. These (NBT-BT-zNN), (z = 0.00, 0.02, 0.04, 0.08) ceramic compositions have been processed by the conventional solid-state mix oxide route, and their dielectric properties, phases, microstructure, and storage energy density were investigated. For z ≤ 0.02, a single perovskite phase was formed, while for z = 0.04, a secondary phase Bi_2_Ti_2_O_7_ also developed. The highly dense microstructural feature appeared for each ceramic. The high energy storage density W_s_ ∼ 3.2 J/cm^3^ and recoverable energy W_rec_ ∼ 2.01 J/cm^3^ with an efficiency of 62.5% were achieved for the composition with the z content of 2 mol% at an electric field of 250 kV/cm, which is a worthy opening in the developments of high-temperature-sustainable ceramic materials for dielectric capacitors.

## Data Availability

The raw data supporting the conclusion of this article will be made available by the authors, without undue reservation.
